# c-myc regulates the sensitivity of breast cancer cells to palbociclib via c-myc/miR-29b-3p/CDK6 axis

**DOI:** 10.1038/s41419-020-02980-2

**Published:** 2020-09-15

**Authors:** Wenfei Ji, Wenwen Zhang, Xin Wang, Yaqin Shi, Fang Yang, Hui Xie, Wenbin Zhou, Shui Wang, Xiaoxiang Guan

**Affiliations:** 1 Department of Oncology, The First Affiliated Hospital of Nanjing Medical University, Nanjing, China; 2 Department of Oncology, Nanjing First Hospital, Nanjing Medical University, Nanjing, China; 3 Medical School of Nanjing University, Nanjing, China; 4 Department of Breast Surgery, The First Affiliated Hospital of Nanjing Medical University, Nanjing, China

**Keywords:** Breast cancer, Prognostic markers

## Abstract

Palbociclib, a CDK4/6 inhibitor, has been granted accelerated approval by US FDA for hormone receptor-positive HER2-negative metastatic breast cancer. To determine potential biomarkers of palbociclib sensitivity to assist in patient selection and clinical development, we investigated the effects of palbociclib in a panel of molecularly characterized breast cancer cell lines. We quantified palbociclib sensitivity and c-myc expression in 11 breast cancer cell lines, 124 breast cancer samples, and The Cancer Genome Atlas database. We found non-TNBC subtypes were more sensitive to palbociclib than TNBC. Activation of c-myc led to differential palbociclib sensitivities, and further inhibition of c-myc enhanced palbociclib sensitivity. Moreover, we identified for the first time a c-myc/miR-29b-3p/CDK6 axis in breast cancer that could be responsible for c-myc-induced palbociclib insensitivity, in which c-myc activation resulted in downregulation of miR-29b-3p, further activated CDK6 and inhibited cell-cycle arrest at G_1_ phase. Moreover, downregulated (inactived) c-myc-induced oncogenic addiction could increase palbociclib efficacy, using both Xenograft model and patient-derived tumor xenograft (PDTX) model. Our finding extends the concept of combined blockade of the CDK4/6 and c-myc signaling pathways to increase palbociclib sensitivity, making c-myc a promising biomarker for palbociclib sensitivity in breast cancer.

## Introduction

Breast cancer is the most common malignancy, the second most common cause of cancer deaths among women^1^. Great advances have been made in the understanding of this malignancy, and several molecular characteristics of breast cancer have been identified^2,3^. Such a molecular understanding has paved the way for the treatment of breast cancer by targeting specific pathogenic molecular alterations. For example, dysregulation of cell-cycle control has been implicated in human malignancies, including breast cancer^4^, and research on cell-cycle control has allowed identification of attractive targets for novel breast cancer therapeutics. Palbociclib is an orally active and highly selective inhibitor of CDK4 and CDK6 kinases, which could block the phosphorylation of retinoblastoma (Rb), subsequently preventing progression of the cell cycle from G_1_ into the S phase in the Rb-positive cells of various tumor types^5–7^.

Preclinical evidence indicated that palbociclib could cause tumor regression, leading to a net decrease in tumor burden^5^. Based on results of the randomized clinical trials, palbociclib has shown impressive progression-free survival (PFS) improvement, when combined with an endocrine treatment in patients who are both endocrine sensitive and endocrine resistant. The trials, known as PALOMA-1 and PALOMA-2, compared palbociclib plus letrozole with letrozole alone in patients who had not previously received endocrine therapies, and the result showed that a longer PFS in the palbociclib-letrozole group^8,9^. Similarly, in the PALOMA-3 trial, for both HR-positive and HER2-negative advanced breast cancer patients who had previously received endocrine therapies, palbociclib plus fulvestrant also resulted in a longer PFS than fulvestrant alone^10^. On the basis of these intriguing results, palbociclib received the FDA-accelerated approval for luminal HER2 negative breast cancer, both as an initial endocrine-based therapy for metastatic breast cancer and in combination with fulvestrant after progression, following an endocrine therapy for advanced breast cancer.

An intact or overactive Rb pathway occurs more frequently in the luminal or HER2-amplified breast cancer. Preclinical evidence demonstrated that these breast cancer subtypes were more sensitive to CDK4/6 inhibitors^11^, which provides the basis for the application of CDK4/6 inhibitors in these tumors. Besides, combination of CDK4/6 inhibitors and other small molecule inhibitors are also explored in hormone receptor-positive breast cancer, including BCL2 inhibitor ABT-199 (venetoclax)^12^, FGFR tyrosine kinase inhibitor (TKI) lucitanib^13^, mTORC1/2 inhibitors^14^, PI3K inhibitors^15^, and AR antagonist enzalutamide^16^. In contrast, triple-negative breast cancer (TNBC), has been considered less likely to respond to CDK4/6 inhibitors^17^. The Rb loss might partially explain the intrinsic resistance to CDK4/6 inhibitors in TNBC^18^; however, molecular alterations associated with the resistance to CDK4/6 inhibitors in this aggressive BC subtype have not been thoroughly elucidated yet. To better elucidate other potential molecular alterations that contributes to the difference in the sensitivity to palbociclib between luminal or HER2-positive breast cancer and triple-negative breast tumors, we therefore explored the effects of palbociclib in a panel of human breast cancer cell lines representative of different breast cancer subtypes.

## Results

### TNBC expressed a high level of c-myc and insensitive to palbociclib than non-TNBC

**Fig. 1:**
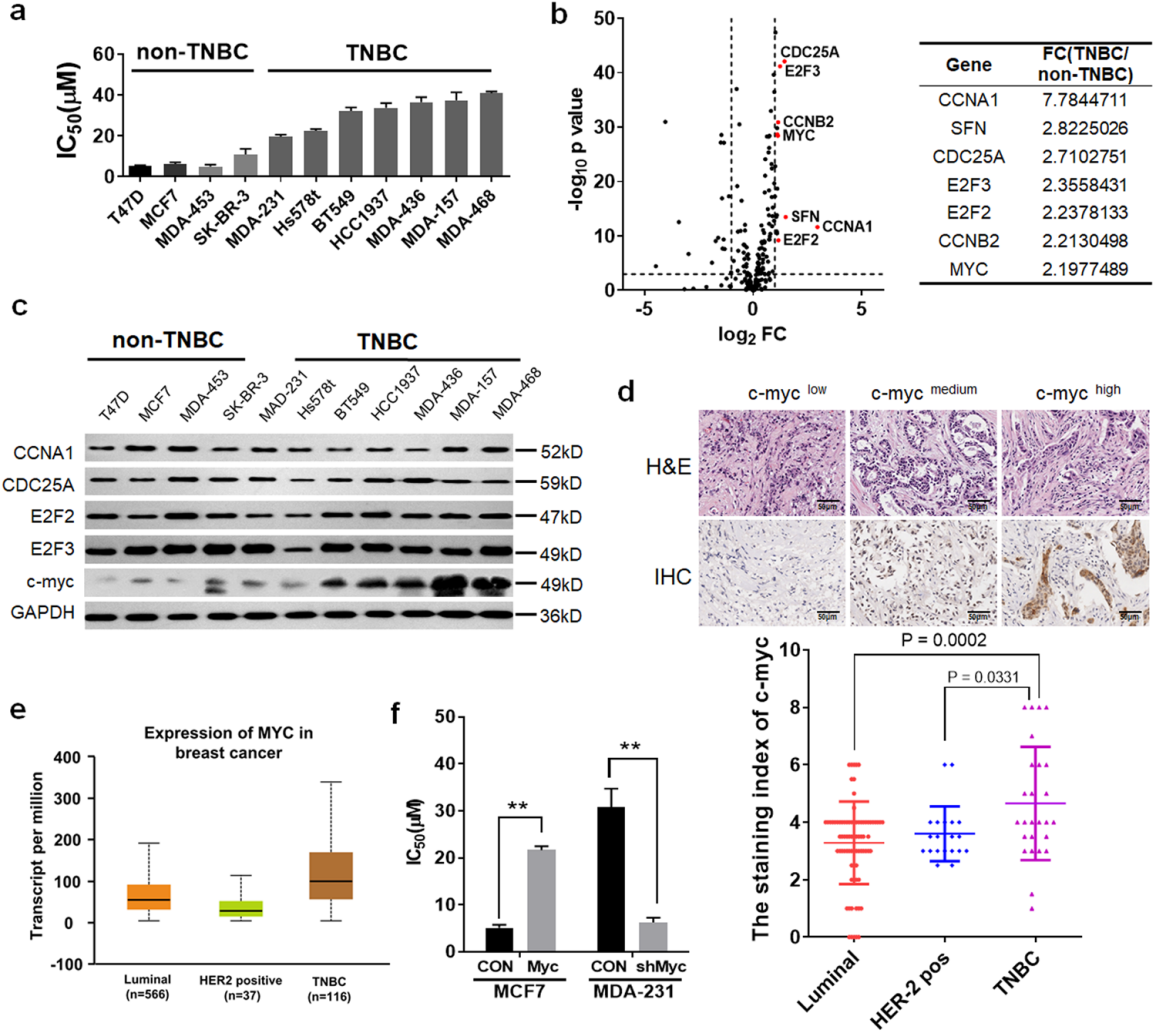
TNBC expressed a high level of c-myc and is insensitive to Palbociclib than non-TNBC. **a** Half-maximal inhibitory concentration (IC_50_) of palbociclib for 11 human breast cancer cell lines were calculated based on the results of CCK-8 assay. **b** Left shows volcano plot comparing the *P* value versus fold change for genes from TNBC relative to non-TNBC patients. The vertical lines correspond to 2.0-fold up and down, and the horizontal line represents a *P* value of 0.001. Genes labeled in red represent the significantly expressed genes. Right illustrates the top 10 highly expressed genes in TNBC compared to non-TNBC patients. **c** Western blot analysis of lysates from 11 human breast cancer cell lines. **d** Up: representative immunohistochemical staining of c-myc low, medium, or high expression. Down: expression of c-myc by immunohistochemical staining in 124 breast cancer samples in tissue microarrays. **e** C-myc mRNA expression in luminal (*n* = 566), HER2 positive (*n* = 37), and triple-negative (*n* = 116) breast cancer samples from The Cancer Genome Atlas (TCGA) database. **f** IC_50_ values for MCF-7 cell transfected with c-myc plasmid and MDA-MB-231 cell transfected with c-myc shRNA treated with palbociclib for 48 h. Representative images and data based on three independent experiments. Error bars indicate mean ± standard deviation.

A panel of 11 human breast cancer cell lines representing of luminal, HER2-positive breast cancer, or TNBC were treated with Palbociclib. The calculated IC_50_ for each of the cell lines and their molecular classification were determined (Fig. 1a). Among these 11 breast cancer cell lines, the luminal or HER2-positive ones were the most sensitive subtypes, whereas TNBC were the most resistant cell lines. Next, we investigated the expression of genes implicated in cell cycle and ER signaling from The Cancer Genome Atlas (TCGA) datasets containing 423 breast cancer cases. A set of 223 genes was identified, and their expression levels were further evaluated between TNBC and non-TNBC patients (*P* < 0.05 and fold change > 1.5). The top highly expressed genes are indicated in Fig. 1b. Given that palbociclib blocks cell cycle at G_1_/S phase, we focused on genes that could critically implicate in the G_1_/S transition. Five of these biomarkers (CCNA1, CDC25A, E2F2, E2F3, and c-myc) with significantly altered expression in TNBC cases were selected. Among these, c-myc, as a potential activator of oncogenic transcription programs, was strongly expressed in TNBC cell lines, but less expressed in luminal or HER2-positive cell lines (Fig. 1c, and Supplementary Fig. 1a–d). Next, we examined the expression levels of c-myc by using immunohistochemical staining in 124 breast cancer tissue microarrays (Fig. 1d, Supplementary Table 1). We found c-myc expression was indeed more enriched in TNBC patients (Fig. 1d, e). Based on the aforementioned findings, we interrogated whether the overexpression of c-myc, which was differentially expressed among breast cancer subtypes, might play an important role in palbociclib insensitivity in TNBC patients. Thus, we further evaluated the effect of c-myc inhibition in palbociclib sensitivity on MCF-7 and MDA-MB-231 cells. As shown in Fig. 1f, the inhibition of c-myc sensitized cells to CDK4/6 inhibitor palbociclib in MDA-MB-231 cells, while c-myc-overexpressed MCF-7 cells showed resistance to palbociclib. Taken together, our data suggest that c-myc is expressed at higher levels in TNBC and may be a feasible target to induce palbociclib activity.

### Inhibition of c-myc expression induces palbociclib sensitivity

**Fig. 2:**
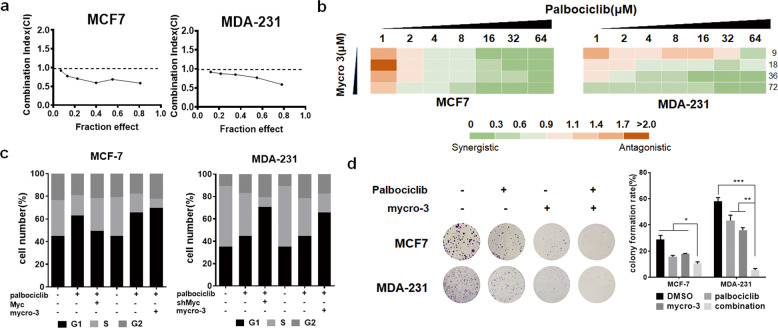
Inhibition of c-myc expression induces palbociclib sensitivity. **a** MCF-7 and MDA-MB-231 cells were treated with the combination of palbociclib and mycro-3. The dose radio of palbociclib and mycro-3 was 1:1. Cell viability was investigated by Cell Counting kit-8 assay. CI vs. Fa plot (combination index vs. fraction affected) for cell viability data are shown. The CI values were calculated by Compusyn software. CI values below 1 are considered to have a synergistic interaction. **b** MCF-7 and MDA-MB-231 cells were treated with 1–64 µM palbociclib combined with a constant dose of mycro-3 (9, 18, 36, 72 µM) for 48 h. CI values calculated by Compusyn software are shown. **c** Cell-cycle analysis of MCF-7 and MDA-MB-231 cells 48 h after treatment with the indicated drugs, Myc plasmid, or shMyc transfection. **d** Colony formation assays of MCF-7 and MDA-MB-231 cells 14 d after treatment with DMSO or the indicated drugs. Colonies were counted and captured. Representative images and data based on three independent experiments. Error bars indicate mean ± standard deviation.

Next, we focused on the role of c-myc expression in the palbociclib sensitivity. Given the benefits associated with targeted cancer therapies (Fig. 1f), we sought to identify if mycro-3, a novel and selective c-myc inhibitor, might be effective in breast cancer cells. We used a fixed ratio model of combination therapy to further study the synergistic effect of mycro-3 and palbociclib. We utilized the combination ratio (1:1) and then calculated the combination index (CI). The combination index vs. fraction affected plot (CI vs. Fa plot) showed that all the CI values in both MCF-7 and MDA-MB-231 cells were below 1, which indicates that the combination of these two drugs had a significant synergistic effect (Fig. 2a). We also treated cells with increasing concentrations of mycro-3 and palbociclib, and both two cell lines exhibited additive to synergistic effects (Fig. 2b). In addition, we also found that combination of palbociclib and c-myc inhibition (mycro-3 or shMYC transfection) could induce synergistic growth inhibition, increased sub-G_1_ cell-cycle population, and cell migration (Fig. 2c, d and Supplementary Fig. 2). At the same time, c-myc overexpression attenuated the G_1_ cell-cycle arrest of palbociclib in MCF-7 cells (Fig. 2c). Collectively, our data suggest that strategies of inhibiting c-myc could suppress cancer progression and increased palbociclib sensitivity.

### Palbociclib-induced miR-29b-3p is negatively regulated by c-myc

**Fig. 3:**
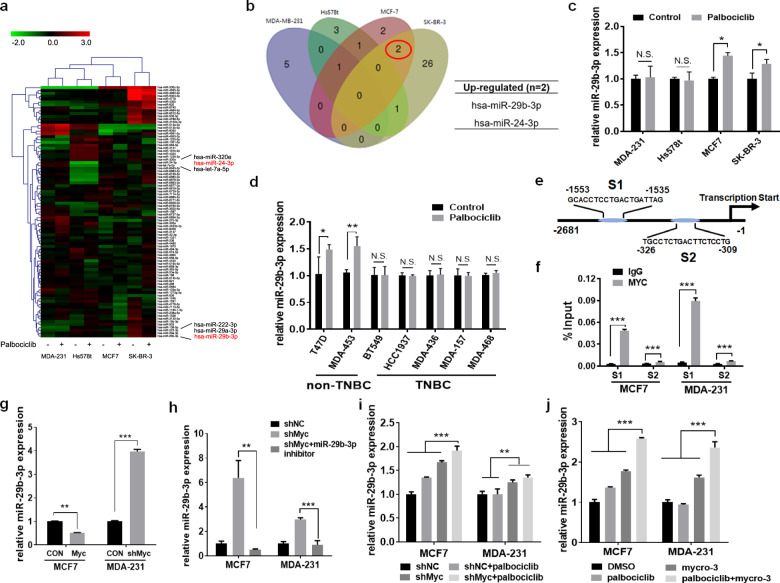
Palbociclib-induced miR-29b-3p is negative regulated by c-myc. **a** A miRNA microarray was performed to detect differentially expressed miRNAs of MDA-MB-231, Hs578t, MCF-7, and SK-BR-3 cells treated with or without 4 μM palbociclib for 48 h. The green in the legend represents downregulation, and the red represents upregulation (>1.5-fold change in expression, *P* < 0.05). **b** Venn diagrams for the number of differentially expressed miRNAs among four breast cancer cell lines after the treatment of palbociclib for 48 h. **c** Validation by quantitative real-time PCR (qRT-PCR) of miR-29b-3p in the four breast cancer cells treated with or without palbociclib. **d** miR-29b-3p in the other seven breast cancer cells treated with or without palbociclib were analyzed by qRT-PCR. U6 was used as an endogenous control for miRNA analysis. **e** Schematic model of c-myc binding sites in S1 promoter and S2 promoter of miR-29b-3p gene by bioinformatic analysis. The sites and sequences of three binding sites were indicated in model scheme. **f** Binding of c-myc in MCF-7 and MDA-MB-231 cells to the miR-29b-3p promoter region was analyzed by CHIP-qPCR. **g** qRT-PCR analysis of miR-29b-3p expression in MCF-7 and MDA-MB-231 cells after overexpression or knockdown of c-myc. Expression of miR-29b-3p in MCF-7 and MDA-MB-231 cells transfected with NC shRNA or c-myc shRNA treated with **h** miR-29b-39 inhibitor or **i** palbociclib was analyzed by qRT-PCR. **j** Expression of miR-29b-3p in MCF-7 and MDA-MB-231 cells following treatment with the indicated drugs was measured by qRT-PCR. Error bars indicate mean ± standard deviation.

MicroRNAs (miRNAs) play crucial roles in regulating cancer biology, including proliferation, apoptosis, and drug resistance^19^. Our previous studies have identified several miRNAs associated with the sensitivity of breast cancer to multiple drugs, including tamoxifen^20^, olaparib^21^, saracatinib^22^, and lapatinib^23^. To investigate whether miRNAs were associated with palbociclib sensitivity, we used microarray to establish differential miRNA expression profiles between palbociclib-sensitive cells and palbociclib-insensitive cells after palbociclib treatment. The hierarchical clustering of differential expressed miRNAs was illustrated in Fig. 3a. Differential expression of two miRNAs (miR-29b-3p and miR-24-3p) between palbociclib-sensitive cells and palbociclib-insensitive cells was detected. Both two miRNAs showed upregulation in MCF-7 and SK-BR-3 cells after treatment with palbociclib, whereas this upregulation was not observed in MDA-MB-231 and Hs578t cells (Fig. 3b). To validate these microarray results, expression of these two miRNAs were studied in four cell lines using the qRT-PCR analysis. Of these two miRNAs, only miR-29b-3p was confirmed to be slightly but significantly upregulated in both MCF-7 and SK-BR-3 cells (Fig. 3c and Supplementary Fig. 3a). Furthermore, we examined miR-29b-3p expression in multiple breast cancer cell lines after palbociclib treatment. The luminal or HER2-positive cell lines had increased expression levels of miR-29b-3p, while TNBC cell lines did not have significant change of miR-29b-3p expression levels, which was consistent with previous findings (Fig. 3d). Conclusively, our data supports a role of miR-29b-3p in regulating palbociclib sensitivity in luminal or HER2-positive cell lines.

On the basis of previous findings, aberrant c-myc activation may underlie the differences of palbociclib sensitivity among breast cancer subtypes. Moreover, miR-29b-3p, known to suppress tumor progression in various tumor types^24–26^, was found to be upregulated in palbociclib-sensitive luminal or HER2-positive breast cancer cells after palbociclib treatment. With this framework, we hypothesized that c-myc interacts with miR-29b-3p to regulate palbociclib sensitivity. Therefore, we used bioinformatics analysis to predict transcription factor binding sites in miR-29b-3p promoters, and it appears that both S1 and S2 promoters have the putative binding sites for c-myc (Fig. 3e). The chromatin immunoprecipitation (ChIP) analysis with the anti-c-myc antibody revealed that c-myc protein bound to the miR-29b-3p promoter (Fig. 3f). Further silencing c-myc increased the levels of both pre-miR-29b-3p and mature miR-29b-3p, while c-myc overexpression decreased both pre-miR-29b-3p and mature miR-29b-3p expression levels. Those data indicated that miR-29b-3p was regulated at the transcriptional level in this c-myc-mediated signaling (Fig. 3g and Supplementary Fig. 3b). Moreover, we also found that miR-29b-3p inhibitor decreased the elevated miR-29b-3p level but enhanced by the silence of c-myc (Fig. 3h). Interestingly, palbociclib treatment could more significantly increase the miR-29b-3p expression in cells transfected with the shMYC plasmid (Fig. 3i). Besides, higher levels of miR-29b-3p were also observed in cells treated with the combination of palbociclib and mycro-3, compared with monotherapy (Fig. 3j). Taken together, our data suggest that c-myc could negatively regulate miR-29b-3p expression by binding to the promoter region of miR-29b-3p.

### miR-29b-3p inhibits breast cancer cell growth and increases sensitivity to palbociclib

**Fig. 4:**
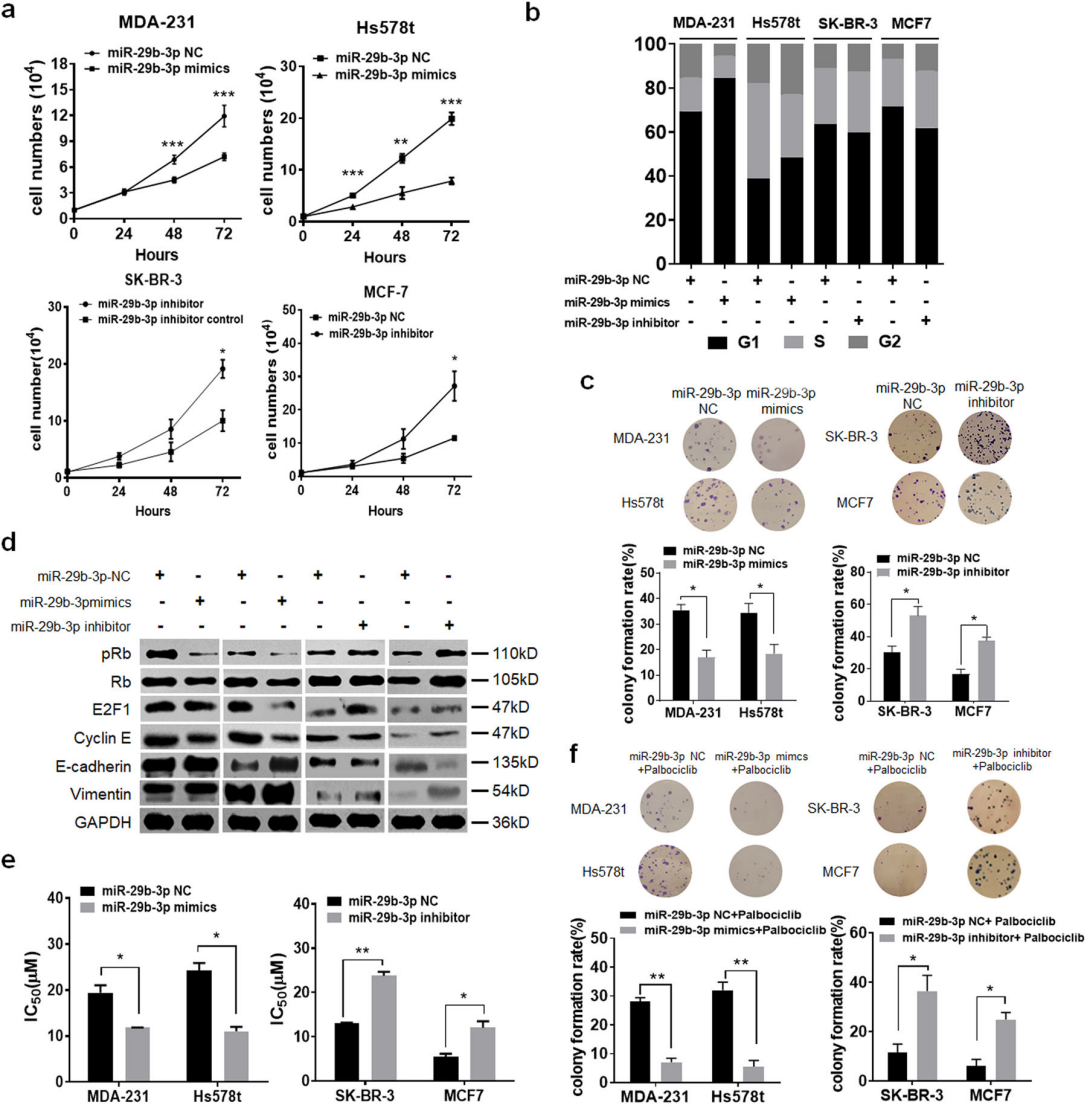
miR-29b-3p inhibits breast cancer cell growth and increases sensitivity to palbociclib. MDA-MB-231 and Hs578t cells were transfected with miR-29b-3p mimics or miR-29b-3p NC. SK-BR-3 and MCF-7 cells were transfected with miR-29b-3p inhibitor or miR-29b-3p. Then **a** cell viability, **b** cell cycle, and **c** colony formation were performed. **d** Western blot analysis of lysates from the four breast cancer cells after transfected. **e** IC_50_ of palbociclib for the four breast cancer cells after transfected. **f** Colony formation assays of the four breast cancer cells following the indicated transfections treated with palbociclib.

We next investigated the role of miR-29b-3p in breast cancer cells. First, we detected the expression levels of miR-29b-3p across the four cell lines (Supplementary Fig. 4a). Thus, we used miR-29b-3p mimics in MDA-231 and Hs578t cells, and miR-29b-3p inhibitor in SK-BR-3 and MCF-7 cells (Supplementary Fig. 4b, c). We found the miR-29b-3p-overexpressing cells exhibited significantly increased cell viability and proportion of cells arrested in the G_1_ cell-cycle phase (Fig. 4a–c). Consistent with its role of accelerating cell-cycle arrest, miR-29-3p inhibited Rb phosphorylation and decreased E2F1 expression (Fig. 4d). We further examined the effect of miR-29-3p in epithelial–mesenchymal transition (EMT) and cell migration process. We found that gain of miR-29-3p was sufficient to inhibit an EMT with significantly more E-Cadherin (Fig. 4d). Results from cell migration assays demonstrated a decreased cell migration after the transfection of miR-29-3p (Supplementary Fig. 4d, e). Furthermore, reducing miR-29-3p levels promoted cell proliferation, cell-cycle progression, colony formation, and cell migration (Fig. 4a–d). We next wanted to know whether miR-29b-3p overexpression would confer the sensitivity to CDK4/6 inhibition. We treated miR-29b-3p-transfected MDA-MB-231 and Hs578t cells with palbociclib. Indeed, cell sensitivity to palbociclib was more enhanced in miR-29b-3p-overexpressing cells (Fig. 4e). Similarly, loss of miR-29b-3p in MCF-7 and SK-BR-3 cells induced the resistance to palbociclib treatment (Fig. 4e). The concept of increasing palbociclib sensitivity by miR-29b-3p was further confirmed by colony formation assay (Fig. 4f). In conclusion, these data support that miR-29b-3p may inhibit breast cancer cell growth and increase sensitivity to palbociclib.

### miR-29b-3p negatively regulates CDK6 expression

**Fig. 5:**
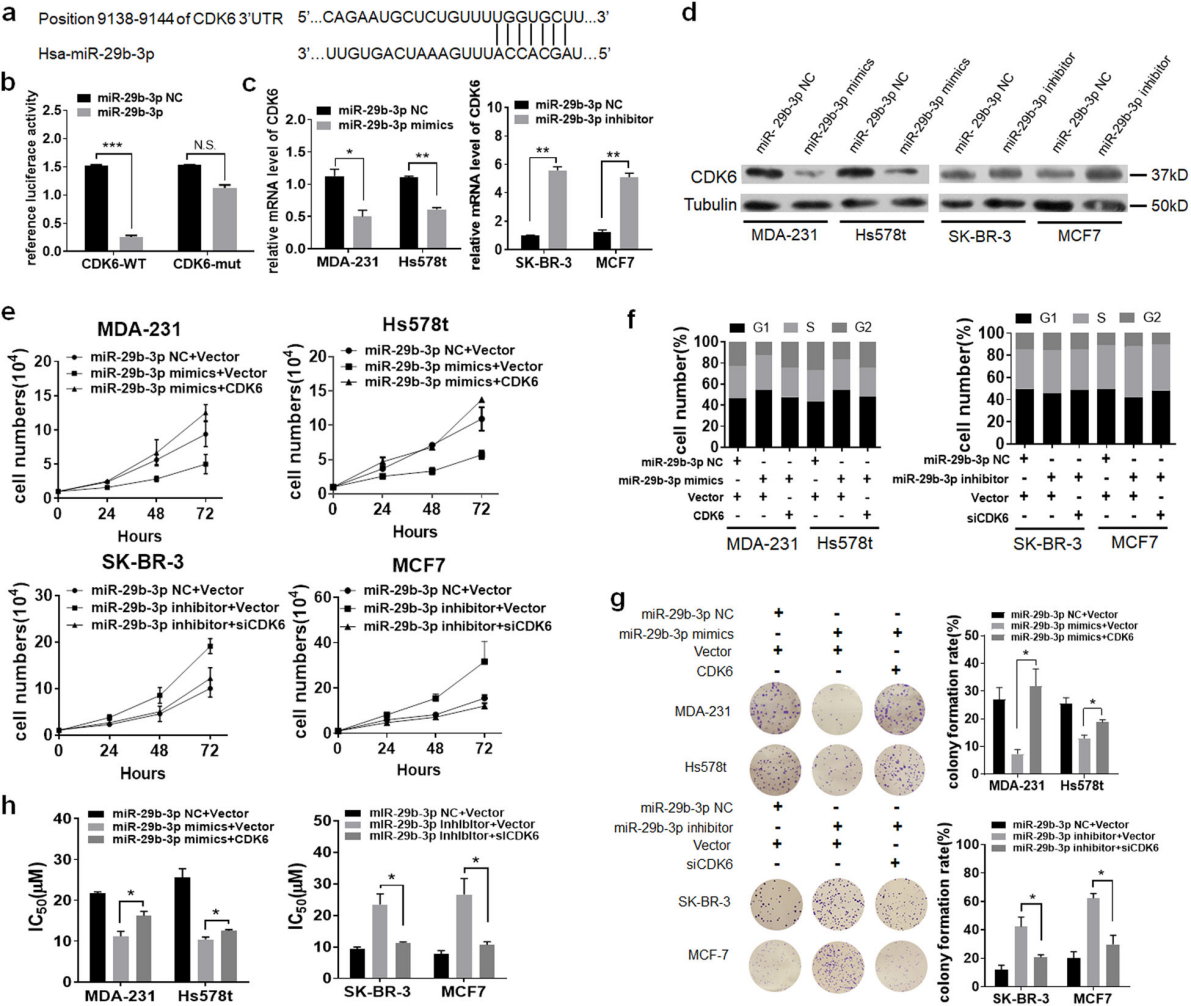
miR-29b-3p negatively regulates CDK6 expression. **a** Both Targetscan and miRDB tools showed schematic representation of putative binding site for miR-29b-3p in 3′-UTR of CDK6. **b** Luciferase reporter plasmids containing wild-type or mutant 3′-UTR of CDK6 was transfected with either miR-29b-3p mimics or a control miRNA into HEK293T cells. **c** qRT-PCR analysis of CDK6 expression in MDA-MB-231, Hs578t, SK-BR-3, and MCF-7 cells transiently transfected with either miR-29b-3p mimics or a control miRNA. **d** Western blot analysis detected the expression of CDK6 after knockdown or overexpression of miR-29b-3p. MDA-MB-231 and Hs578t cells were transfected with miR-29b-3p mimics or overexpression plasmid of CDK6. SK-BR-3 and MCF-7 cells were transfected with miR-29b-3p inhibitor or siCDK6 plasmid. Then **e** cell viability, **f** cell cycle, and **g** colony formation were performed. **h** IC_50_ of palbociclib for the four breast cancer cells after transfected as indicated. Error bars indicate mean ± standard deviation.

We further explored the molecular mechanisms responsible for the multiple functions of miR-29b-3p. Potential target genes of miR-29b-3p were predicted by using tools including Targetscan and miRDB. The critical regulator of G_1_/S transition signaling, CDK6, predictively contains putative binding sites for miR-29b-3p in the 3′-untranslated region (UTR) (Fig. 5a). Luciferase activity of the reporter linked with CDK6 3′-UTR was repressed by transfection of miR-29b-3p mimics, and mutations of the 3′-UTR of *CDK6* completely abolished the repressive effects (Fig. 5b). Moreover, the mRNA expression levels of *CDK6* were remarkably reduced after transfection of miR-29b-3p mimics in both MDA-MB-231 and Hs578t cells, which were reversely upregulated following miR-29b-3p inhibition in SK-BR-3 and MCF-7 cells (Fig. 5c). Consistently, western blot analysis confirmed that the CDK6 protein levels were negatively modulated by miR-29b-3p (Fig. 5d). These findings indicate that miR-29b-3p could negatively modulate CDK6 expression by directly targeting its 3′-UTR.

Next, the role of CDK6 in miR-29b-3p-mediated effects was evaluated. We demonstrated that overexpression of CDK6 in miR-29b-3p-transfected MDA-MB-231 and Hs578t cells attenuated the inhibitory effect of miR-29b-3p on multiple cancer-related functions, including cell growth, cell G_1_/S transition, and cell migration. Cells transfected with miR-29b-3p inhibitor induced cell growth and migration, as well as G_1_/S transition, whereas silencing CDK6 in the pre-transfected cells could antagonize the function of downregulating miR-29b-3p in SK-BR-7 and MCF-7 cells (Fig. 5e–g and Supplementary Fig. 5), suggesting that the biological effects of miR-29b-3p could be attributable to the altered CDK6 signaling. Consistently, CDK6 overexpression could reduce cell growth, migration and G_1_/S transition in miR-29b-3p-overepressing MDA-MB-231 and Hs578t cells (Fig. 5e–g and Supplementary Fig. 5). In addition, transfection of miR-29b-3p mimics enhanced sensitivity to CDK4/6 inhibition palbociclib, while upregulation of CDK6 could reverse this sensitivity (Fig. 5h). Taken together, these results disclosed that miR-29b-3p negatively regulates CDK6 expression.

### Inhibition of c-myc sensitizes breast cancer cells to palbociclib in vivo

**Fig. 6:**
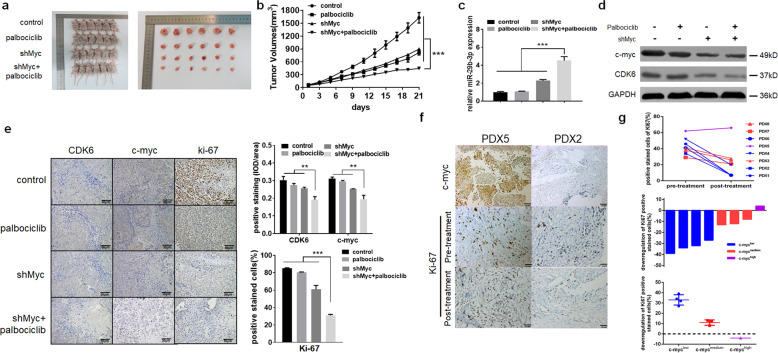
Inhibition of c-myc sensitizes breast cancer cells to palbociclib in vivo. Empty vector or shMyc were transfected into MDA-MB-231 cells, which were injected into nude mice, respectively. Mice bearing MDA-MB-231 xenografts with a tumor volume of 100 mm^3^ (6 for each group) was then randomly grouped and treated with vehicle (orally), or palbociclib at a dose of 100 mg/kg twice a week by oral gavage. **a** Visualization of the mice and tumors, after 21 days of initial treatment. **b** Tumor volumes were calculated every 3 days. **c** The qRT-PCR was performed to detect the average expression miR-29b-3p in xenograft tumors. **d** Western blot analysis of c-myc and CDK6 in xenograft tumors. GAPDH was used as an internal control. **e** The tumors were removed from the mice in 21 days after drug treatment ended, and immunohistochemical staining for CDK6, c-myc, and Ki-67 were conducted. Expression ratio of CDK6, c-myc, and ki67 in each of the treatment arms are shown. **f** Immunohistochemical staining of c-myc and Ki-67 in patient-derived tumor xenograft (PDX) model of patient 2 and 5. **g** The positive stained cells of Ki-67 before and after palbociclib treatment in all eight PDX models. Error bars indicate mean ± SD.

To further explore whether c-myc affects the sensitivity of palbociclib to breast cancer cells in vivo, MDA-MB-231 cells stably transfected with sh-c-myc or control vector were inoculated into nude mice. Then the mice were treated with vehicle or palbociclib at a dose of 100 mg/kg twice a week orally. Over a period of three weeks, co-treatment with sh-c-myc and palbociclib significantly inhibited tumor growth, compared with single-agent treatment (Fig. 6a, b). We also observed that inhibition of c-myc enhanced miR-29b-3p level and silence of c-myc plus palbociclib treatment could significantly increase the miR-29b-3p expression (Fig. 6c). In addition, the expression levels of CDK6, c-myc, and Ki-67 were reduced more obviously in co-treatment group by using western blotting and immunohistochemistry staining (IHC staining) (Fig. 6d, e). Therefore, it appears that co-treatment with inhibition of c-myc and palbociclib significantly inhibited tumor growth in mice xenograft model.

Next, we established patient-derived tumor xenograft (PDTX) models using eight tumor tissue samples. Among the eight patients, one was TNBC that had a high expression level of c-myc, and the other seven patients were non-TNBC with a low or medium expression level of c-myc (Supplementary Table 2). A dose of 100 mg/kg palbociclib was then used to treat the PDTX models every day for 7 days, and IHC staining of Ki-67 was performed to further explore the efficacy of palbociclib treatment. As shown in Fig. 6f, there was no change of Ki-67 expression in the PDX5 (TNBC patient), while the expression levels of Ki-67 in the PDX2 (Luminal patient) were significantly decreased after palbociclib treatment. The results of other PDTX models showed the same tendency (Fig. 6g). Taken together, our data suggest that inhibition of c-myc may sensitize breast cancer cells to palbociclib in vivo.

## Discussion

In the present study, we confirmed that compared with luminal or HER2-positive breast cancer cells, TNBC cells exhibited less sensitivity to CDK4/6 inhibition. Furthermore, by analyzing the differentially expressed genes between TNBC and non-TNBC groups, we selected c-myc as a critical factor implicated in G_1_/S transition, and we found that further inhibition of c-myc led to the enhanced palbociclib sensitivity. Moreover, we identified for the first time a c-myc/miR-29b-3p/CDK6 axis that might be responsible for the c-myc-induced palbociclib insensitivity, in which c-myc activation resulted in downregulation of miR-29b-3p, which enhanced CDK6 expression. Collectively, our preclinical work suggests that c-myc plays an important role in palbociclib sensitivity and are indicative as a potential predictor of palbociclib response by which the miR-29b-3p/CDK6 axis is critically involved in the c-myc-driven palbociclib insensitivity.

In PALOMA-2, with a 10-month improvement of PFS as the first-line treatment for advanced HR-positive, HER2-negative breast cancer, when combined with letrozole, palbociclib represents one of the best steps forward in the treatment of luminal breast cancer^9^. Nonetheless, questions remain regarding the molecular mechanism underlying the observed improvement in the treatment. The key change is the identification of predictive indicators and biomarkers for better patient selection. In the palbociclib-letrozole group, the rate of confirmed objective response was 55.3%, which suggests that nearly 45% of luminal HER2-negative breast cancer patients could not benefit from this combination. Moving forward, identifying response predictors will be essential for a rational use of palbociclib to avoid unnecessary disease progression and treatment-related costs.

C-myc controls for various biological functions, including cell growth and proliferation, differentiation and programmed cell death in aggressive breast cancer^27^. The genomic region, 8q24, which contains the c-myc oncogene locus, has been detected with a frequently amplified rate in the basal-like breast cancer^28,29^. Some emerging data suggest that c-myc overexpression is also implicated in drug resistance in different tumor types^30^. In the present study, we reasoned that c-myc overexpression might be indicative of the sensitivity to CDK4/6 inhibitor. As we hypothesized, we found that both the shRNA-mediated and small molecular inhibitor-induced inhibition of c-myc significantly increased palbociclib sensitivity. Besides, in patient-derived breast tumors, c-myc expression could serve as a predictive marker to assess the response of palbociclib. In fact, during the G_0_-to-S phase progression, an elevated expression of c-myc elicited a shortened G_1_ phase by activating the CDK4/6 activity, thus inducing palbociclib resistance in breast cancer. This led to a hypothesis in therapeutic intervention: for tumors displaying a significant shortening of G_1_ phase, specific targeting of cell-cycle regulators in the G_2_ phase can result in more cell death. An exciting application for this hypothesis was confirmed by Horiuchi et al.^31^, which indicates that aggressive breast tumors with elevated c-myc expression were uniquely sensitive to the CDK1/2 inhibitors. In conclusion, we have shown c-myc as a potential predictor of response for the CDK4/6 inhibition. It is likely that c-myc-overexpressing breast tumors might not be sensitive to the CDK4/6 inhibition but may be more responsive to the CDK1/2 inhibitors. Thus, the detection of c-myc activity has the potential to be translated into potential clinical use. Therefore, we propose that c-myc status will be useful as a predictive biomarker or indicator for the response to CDK4/6 inhibitors for the treatment of breast cancer.

To explore the mechanism of the c-myc signaling in palbociclib sensitivity, we further identified that miR-29b-3p was negatively regulated by c-myc in breast cancer cells. The miR-29 family consists of three members: miR-29a, miR-29b, and miR-29c. Dysregulation of this family has been implicated in various cancers, including leukemia, lung cancer, liver cancer, breast cancer, and melanoma^24–26^. In breast cancer, miR-29b-3p is enriched in luminal breast cancer, and loss of miR-29b may promote metastasis or form a mesenchymal phenotype. Mechanistically, miR-29b is likely to inhibit a network of pro-metastatic regulators implicated in angiogenesis, collagen remodeling, and proteolysis to affect differentiation and epithelial plasticity^32,33^. In the present study, we found that miR-29b-3p was responsible for c-myc signaling-mediated palbociclib resistance. Both our gain- and loss-of-function studies demonstrated that miR-29b-3p dramatically suppressed the ability of breast cancer cells to promote proliferation and palbociclib resistance. Therefore, we have found that miR-29b-3p-mediated suppressive function depended on the repression of the CDK6 signaling. However, in the present study, we only determined the regulation of miR-29b-3p/CDK6 on the c-myc-induced palbociclib insensitivity. Several studies determined that clinical CDK4/6 Inhibitors are more potent against CDK4 than CDK6^34,35^. The role of miR-29b-5p and CDK4 on the palbociclib insensitivity in breast cancer should also be investigated in the future.

**Fig. 7:**
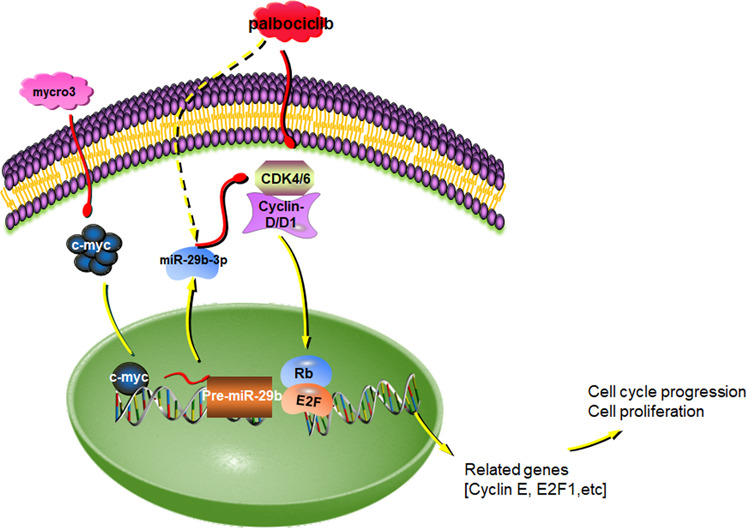
A schematic model of c-myc/miR-29b-3p/CDK6 axis in the regulation of palbociclib sensitivity in breast cancer cells. Mycro3, a selective c-myc inhibitor, inhibit the expression of c-myc. c-myc could negatively regulate miR-29b-3p expression by binding to the promoter region of miR-29b-3p. miR-29b-3p negatively modulate CDK6 expression by directly targeting its 3'-UTR, leding to the enhanced palbociclib sensitivity and induced tumor growth.

Based on the data presented above, we have defined a signaling axis that c-myc downregulates miR-29b-3p to promote activation of CDK6, thus inducing palbociclib resistance in breast cancer. In addition, we have shown that miR-29b-3p may be a tumor suppressor through repressing expression of CDK6, which is strongly involved in palbociclib sensitivity (Fig. 7). These findings demonstrate a predictive relevance in the signaling pathway initiated by c-myc, as we have identified c-myc as a potential predictor of the response to the CDK4/6 inhibition in breast cancer. Our findings also provide the rationale to identify tumors with c-myc overexpression and more predictive response to the combination of CDK4/6 inhibitors and c-myc inhibitors.

## Materials and methods

### Cell lines and culture conditions

The T47D, MCF-7, MDA-MB-453, SK-BR-3, MDA-MB-231, Hs578t, BT549, HCC1937, MB436, MB-157, and MDA-MB-468 cell lines were purchased in 2016–2017 from the Chinese Academy of Science Committee Type Culture Collection Cell Bank (Shanghai, China). Authenticity of these cell lines was done by Chinese Academy of Science Committee Type Culture Collection Cell Bank before purchase by STR DNA typing methodology. MDA-MB-231, MB-157, BT549, HCC1937, SK-BR-3, MDA-MB-453, T47D, and MCF-7 cells were grown in RPMI 1640 (Gibco, USA) supplemented with 10% fetal bovine serum (FBS) (Hyclone USA), Hs578t and MDA-468 cells were grown in DMEM supplemented with 10% FBS, MB436 was cultured in L15 medium with 10% FBS. All culture media contained 100 units/ml of penicillin and 100 units/ml of streptomycin and all cell lines were incubated in a humidified atmosphere with 5% CO_2_ at 37 °C.

### miRNA microarray

MDA-MB-231, Hs578t, SK-BR-3, and MCF-7 cells were treated with or without palbociclib for 48 h. For the miRNA microarray assay, the total RNA was isolated from the four cell lines using the Trizol Reagent (Invitrogen, Carlsbad, CA, USA). Small RNA was extracted by using a mirVana kit (Ambion, Austin, USA) and then labeled with Cy5 fluorescent dyes to hybridize to single-channel microarrays on each chip consisting of 2576 probes for the detection of 1321 human miRNA. The raw data were normalized and adjusted by using GenePix Pro 4.0 software. The miRNA expression in the experimental group (each cell line treated with palbociclib) was compared with that in the control group (each cell line with no treatment), and the fold change was calculated. The accession number for the deposited miRNA sequencing data reported in this paper is GEO: GSE136671.

### Antibodies and agents

For this study, we purchased the following antibodies against CCNA1 (Abcam, ab172317), CDC25A (Abcam, ab92892), E2F2 (Abcam, ab138515), E2F3 (Abcam, ab50917), c-myc (Abcam, ab39688), pRb (S795) (Cell Signaling Technologies, #9301), Rb (Abcam, ab181616), E2F1 (Abcam, ab179445), E-cadherin (Cell Signaling Technologies, #8834), Vimentin (Abcam, ab92547), cyclin E1 (Abcam, ab21142), CDK6 (Abcam, ab151247), Tubulin (Abcam, ab6160), β-actin (Abcam, ab8226), Ki-67 (Cell Signaling Technologies, #9449), mouse IgG (Cell Signaling Technologies, #7076), rabbit IgG (Cell Signaling Technologies, #7074), and GAPDH (Abcam, ab181603). Palbociclib and mycro-3 were obtained from MedChem Express, and both compounds were diluted in DMSO.

### Clinical samples and immunohistochemistry

Tissue microarray containing HBre-Duc140Sur-01 were provided by Outdo Biotech (Shanghai, China), which contained a total of 140 cancer cases of breast cancer and only a single punch for the one patient. Inclusion criteria included female sex, original histological diagnosis of invasive breast carcinoma, validation of the status of ER, PR, and HER2 as well as availability of clinical pathological data. Experiments with tumor tissues were conducted in compliance with the Helsinki Declaration and approved by the Ethics Committee of our hospital. Pathologic staging was performed in accordance with the International Union against Cancer tumor-lymph node-metastasis classification.

One hundred and twenty-four breast tumors were deparaffinized in xylene. Heat-mediated antigen retrieval was fulfilled with citrate buffer (BioGenex Laboratories, San Ramon, CA). Antibody against c-myc was used for immunohistochemistry staining. Antibody staining was visualized with 3,3′-Diaminobenzidine (DAB) (Sigma, D-5637) and hematoxylin counterstain. Analysis of immunohistochemistry was performed as previously described^36–38^.

### Cell viability assay

Cells were plated at the intensity of 4000–6000 cells per well in 96-well plates. The next day, cells were provided with the fresh medium including different concentrations of the indicated agents. After 48-h exposure of the indicated agents, cell survival was assessed with the Cell Counting Kit-8 in accordance with the recommended guideline (KeyGEN Biotech, Nanjing, China). Combination index (CI) values were calculated by using CompuSyn software (ComboSyn, Inc., NJ, USA).

### Quantitative real-time PCR

The total RNA was extracted from cells using the Trizol Reagent (Invitrogen, Carlsbad, CA, USA). For mRNA detection, c-myc and CDK6 mRNA expression was analyzed by the SYBR Premix Ex Taq II (Takara, Japan), following the manufacture’s introductions. Primer pairs were as follows: c-myc, forward 5′-CGTCTCCACACATCAGCACAA-3′ and reverse 5′-CACTGTCCAACTTGACCCCTCTTG-3′; CDK6, forward 5′-ACGTGGTCAGGTTGTTT-3′ and reverse 5′ GAPDH, forward 5′-TGTTGCCATCAATGACCCCTT-3′ and reverse 5′-CTCCACGACGTACTCAGCG-3′. For analysis of miRNA expression, the total RNA from tissues and cells was extracted from cells with the Trizol Reagent (Invitrogen, Carlsbad, CA, USA) according to the manufacture’s protocol and was reverse transcribed using the miRNA cDNA Synthesis kit (Abm Canada Inc., Milton, ON, Canada). The expression level of miR-29b-3p was detected by using RT Master Mix (Abm Canada Inc., Milton, ON, Canada). All the assays were performed in triplicate, and the results were normalized by the U6 expression. The following primers were used: miR-29b-3p, forward 5′-TCAGGAAGCTGGTTTCATATGGT-3′ and reverse 5′- CTCAACTGGTGTCGTGGAGTCGGCAATTCAGTTGAGAACACTGA-3′; pre-miR-29b, forward 5′-TCAGGAAGCTGGTTTCATATGGT-3′ and reverse 5′-CCCCCAAGAACACTGATTTCAA-3′; miR-24-3p, forward 5′-TGCCTACTGAGCTGATATCAGT-3′ and reverse 5′-GAATACCTCGGACCCTGC-3′; U6, forward 5′-CTCGCTTCGGCAGCACA-3′ and reverse 5′-AACGCTTCACGAATTTGCGT-3′; The relative expression of the target genes was determined using the 2^−ΔΔCt^ method.

### Western blot

The total protein was extracted using a RIPA buffer containing the protease and the phosphatase inhibitor cocktails, and protein concentrations were quantified by using a BCA kit (KeyGEN Biotech, Nanjing, China).

### Transfection assay

The siRNA targeting c-myc were transfected into cells by using lipoFilter (Hanheng, Shanghai, China) following the manufacturer’s instructions. Six hours after the transfection, we replaced the transfection with the normal ones. One of the three siRNAs for c-myc with the highest targeting efficiency was chosen for further studies (The targeted siRNA sequences are as follows: siMYC-1: 5′-ACGGAACUCUUGUGCGUAAUU-3′, siMYC-2: 5′-GAACACACAACGUCUUGGAUU-3′, siMYC-3: 5′-AACGUUAGCUUCACCAACAUU-3′, and control siRNA: 5′-UUCUCCGAACGUGUCACGUTT-3′). LV-myc-RNAi (67581-11; 5′-CCGGGAATGTCAAGAGGCGAACACACTCGAGTGTGTTCGCCTCTTGACATTCTTTTTG-3′) was purchased from GENECHEM (Shanghai, China). Cells were infected with viral particles in complete media. Eight hours later, media containing the viruses were washed and replaced with fresh media. Puromycin (Invitrogen, Carlsbad, CA, USA) selection for infected cells was performed for 14 days.

Cells were seeded in a 6-well plate and cultured for 24 h. On the following day, MDA-MB-231 and Hs578t cells were transfected with miRNA mimics (miR-29b-3p mimics, 5′-UAGCACCAUUUGAAAUCAGUGUUCACUGAUUUCAAAUGGUGCUAUU-3′) or negative control oligonucleotide (miR-29b-3p NC, 5′-UUCUCCGAACGUGUCACGUTT-3′) by using the transfection regent Lipofectamine^TM^ 2000 (Invitrogen, CA), according to the manufacturer’s instructions. SK-BR-3 and MCF-7 cells were transfected with miRNA inhibitors (miR-29b-3p inhibitor, 5′-AACACUGAUUUCAAAUGGUGUA-3′) or the NC (miR-29b-3p NC, 5′-CAGUACUUUUGUGUAGUACAA-3′). For the reduction and induction of CDK6 expression. GFP-tagged of human plenti/CDK6 and plenti/siCDK6 plasmid (5′-GAACAGACAGAGAAACCAAACTAACTTTA-3′) were purchased from Abm (Canada Inc., Milton, ON, Canada). Matched controls (plenti-Blank and Scrambled siRNA) were synthesized from Abm (Canada Inc., Milton, ON, Canada). Cells were transfected with plasmids using Lipofectamine™ 2000 (Invitrogen, CA) following the manufacturer’s instructions. Stably transfected cells were selected for 14 days in the presence of 2 μg/ml puromycin (Invitrogen, Carlsbad, CA, USA).

### Cell-cycle analysis

Cells were collected and centrifuged at 1500 rpm for 5 min. The cells were washed with ice-cold PBS and fixed with 70% ethanol overnight at −20 °C. The fixed cells were washed with PBS for 10 min and treated with RNAase A for 30 min followed by incubation with propidium iodide for 30 min at room temperature. The cell cycle in each specific sample were evaluated by the flow cytometer (FACS Calibur, BD Biosciences, USA) following the manufacture’s instruction.

### Colony forming assay

The cells were seeded in six-well plates at 500 cells per well. After incubating for 24 h, cells were treated with indicated drugs for 24 h. After the drug administration, cells were washed with PBS and were incubated in fresh medium for 14 days. The colonies were fixed with 100% methanol and stained with 0.5% crystal violet. Colonies containing over 50 cells were counted as survivors.

### Transwell assay

The cells were transfected with the distinct siRNA, plasmid or the lentivirus before performing the transwell assay. The migration assay was performed by using Corning™ 24-well invasion assay plate (Corning, NY, USA) according to the manufacturer’s instructions, and 2 × 10^4^ cells were seeded in 200 μL serum-free medium in which the indicated compounds were also attached in the upper layer. Then, 800 μL medium containing 10% FBS was injected to the lower chamber. After 24 h for incubation, the cells below the matrix membrane were consider as the migrated cells. After being fixed with the methanol and stained with the crystal violet, the inserts were photographed.

### ChIP assay

Chromatin immunoprecipitation (ChIP) assay was performed using the ChIP Assay kit according to manufacturer instructions. Briefly, 70% confluent MCF-7 and MDA-MB-231 cells were treated with DMSO for 24 h and then fixed in 1% formaldehyde for 15 min. Cells were lysed, and nuclei were pelleted by centrifugation. Nuclei were resuspended and sonicated on ice using a sonicator to shear the cross-linked DNA to an average length of 200–1000 bp and centrifuged at 12,000 rpm to remove insoluble material. Sheared chromatin was immunoprecipitated with 3 µg of anti-c-myc or control IgG antibody overnight at 4 °C. The immunoprecipitated DNA was treated with RNase for 30 min at 37 °C and then treated with proteinase K for an hour at 45 °C. DNA was then purified with the Qiagen PCR Purification Kit (Qiagen). The purified DNA was analyzed by Quantitative real-time PCR. Primers sequences used for the quantitative real-time PCR were as follows: S1, forward 5′-GCACCTCCTGACTGATTAG-3′ and reverse 5′-TTTCTCCGTCTGACTTTCC-3′; S2, forward 5′-TGCCTCTGACTTCTCCTG-3′ and reverse 5′-GTTGCCCAGACAAAGGTT-3′.

### Luciferase assay

The CDK6-3′-UTR-reporter assay was performed with 293T cells that were cultured in 24-well plates, which were then then co-transfected with 100 ng of the wild-type 3′-UTR reporter or mutant 3′-UTR reporter and 5 pmol of either the miR-29b-3p mimics or the miR-29b-3p NC. After 24 h of incubation, firefly and Renilla luciferase activities of the cell lysates were measured by using the Dual-Luciferase Reporter Assay System (Promega, WI, USA).

### Tumor xenograft experiments and immunohistochemistry

BALB/c nude mice (female, age 4–6 weeks) were purchased from Jinling Hospital (Nanjing, China) and maintained in a maintainer under controlled temperature and humidity, in a 12-h light-dark cycle, with sterile food and water ad libitum. Animal studies were performed in accordance with the Guide for the Care and Use of Laboratory Animals, and all experimental protocols were approved by the Animal Ethics Committee. A total of 24 mice were divided into two groups, empty or LV-c-myc-RNAi transfected- MDA-MB-231 cells (5 × 10^6^) were injected subcutaneously into the right anterior inguinal region of the mice. Tumor size was measured using callipers, and tumor volume was calculated according to the following equation: (long axis × short axis^2^)/2. When the average tumor volume reached ~100 mm^3^, 12 mice in each group are randomized into 2 groups. The mice were treated with vehicle (orally), or palbociclib at a dose of 100 mg/kg twice a week by oral gavage (p.o.). Tumors were measured once every 3 days. Tumors were taken 8 h after the last administration for the following experiments. Following deparaffinization, sections were rehydrated and subjected to antigen retrieval using citrate buffer (BioGenex, USA). The slides were incubated with the indicated antibodies (1:200) at 4 °C overnight. The following steps were performed as previously described. The percentages of positive cells were scored as previously described^37^. The IODs of each image were measured and counted by using Image-Pro Plus v6.0 software (Media Cybernetics Inc, Bethesda, MD, USA).

### Patient-derived tumor xenograft (PDTX) experiments

A total of eight breast tumor tissues were collected from The First Affiliated Hospital of Nanjing Medical University (Nanjing, China) in August 2018. No patients received chemotherapy or radiotherapy prior to the surgery. All patients participated in this study provided a written informed consent for the study. The experiments were approved by the Ethic Committee of the hospital and were conducted in compliance with the Helsinki Declaration. Disease histology was determined in accordance to the criteria of the World Health Organization. Pathologic staging was performed in accordance to the current International Union against Cancer tumor-lymph node-metastasis classification. The patient-derived tumor xenograft (PDTX) experiments were performed as previously described^39^.

### Statistical analysis

All statistical tests were conducted with GraphPad Prism version 8.0. Data were analyzed using a Student’s *t*-test and presented as mean ± SD of three independent experiments unless stated otherwise.

## Supplementary information

Supplementary tables and figure legends

Supplementary figure 1

Supplementary figure 2

Supplementary figure 3

Supplementary figure 4

Supplementary figure 5
