# Supplementary material for: c-myc regulates the sensitivity of breast cancer cells to palbociclib via c-myc/miR-29b-3p/CDK6 axis
Source: Cell Death Dis. 2020 Sep 15;11(9):760. doi: 10.1038/s41419-020-02980-2 (PMC7493901; doi:10.1038/s41419-020-02980-2)
Supplement: Supplementary file 1 — Supplementary tables and figure legends [file 41419_2020_2980_MOESM1_ESM.doc]

**c-myc regulates the sensitivity of breast cancer cells to palbociclib via c-myc/miR-29b-3p/CDK6 axis**

Wenfei Ji, Wenwen Zhang, Xin Wang, Yaqin Shi, Fang Yang, Hui Xie, Wenbin Zhou, Shui Wang, Xiaoxiang Guan

**Supplementary Table 1. The clinical and pathological characteristics of patients in tissue microarray**

| **Characteristics** | **Number of patients** | **%** |
| --- | --- | --- |
| **Tumor subtypes** |  |  |
| Luminal | 78 | 62.9 |
| HER2-positive | 20 | 16.1 |
| TNBC | 26 | 21 |
| **Age** |  |  |
| <35 | 6 | 4.8 |
| 35-49 | 43 | 34.7 |
| 50-59 | 32 | 25.8 |
| ≥60 | 43 | 34.7 |
| **Nodal status** |  |  |
| 0 | 45 | 36.3 |
| 1-3 | 38 | 30.6 |
| ≥4 | 37 | 29.8 |
| Unknown | 4 | 3.2 |
| **Histological grade** |  |  |
| <II | 34 | 27.4 |
| II-III | 89 | 71.8 |
| ≥III | 1 | 0.8 |
| Unknown | 0 | 0 |

**Supplementary Table 2. The list of patients from whom the tumor tissues have been taken for patient-derived xenografts (PDX) establishment**

| No. | Sex | Age | ER | PR | Her-2 | Ki-67(%) | c-myc |
| --- | --- | --- | --- | --- | --- | --- | --- |
| 1 | Female | 37 | ++ | + | - | 46 | low |
| 2 | Female | 65 | +++ | ++ | ++ | 34 | low |
| 3 | Female | 40 | ++ | ++ | - | 41 | medium |
| 4 | Female | 36 | +++ | ++ | - | 52 | low |
| 5 | Female | 52 | - | - | - | 62 | high |
| 6 | Female | 55 | +++ | ++ | + | 41 | low |
| 7 | Female | 77 | +++ | +++ | - | 29 | medium |
| 8 | Female | 49 | +++ | +++ | - | 38 | medium |

**Supplementary figure legends**

**Supplementary figure 1 related to figure 1. TNBC expressed a high level of c-myc and is insensitive to Palbociclib than non-TNBC.** Expression levels of (a) CCNA1, (b) CDC25A, (c) E2F3 and (d) E2F2 in luminal(n=566), HER-2 positive(n=37) and triple negative(n=116) breast cancer samples from The Cancer Genome Atlas (TCGA) database. (e)The qRT-PCR and (f) western blot analysis were performed to detect c-myc in MCF-7 and MDA-MB-231 cells transfected with c-myc shRNA1, shRNA2, or shRNA3.

**Supplementary figure 2** **related to figure 2.** **Inhibition of c-myc expression induces palbociclib sensitivity.** (a) Western blot analysis of c-myc protein levels in MCF-7 and MDA-MB-231 cells treated with mycro-3. (b) Colony formation assays of MCF-7 and MDA-MB-231 cells transfected with shNC or shMyc after treatment with DMSO or palbociclib. Colonies were counted and captured. (c, d) Transwell experiment for the migration ability of MCF-7 and MDA-MB-231 cells 24h after treatment with shMyc transfection or the indicated drugs. Cells on the bottom surface of the well were counted. Representative images and data based on three independent experiments. Error bars indicate mean ± standard deviation.

**Supplementary figure 3** **related to figure 3.** **Palbociclib-induced miR-29b-3p is negative regulated by c-myc.** (a)The qRT-PCR was performed to detect miR-24-3p in the four breast cancer cells treated with or without palbociclib. (b) miR-29b-3p expression level was detected in the MCF7 and MDA-MB-231 cells after c-myc plasmid or shMyc transfection.

**Supplementary figure 4 related to figure 4.** **miR-29b-3p inhibits breast cancer cell growth and increases sensitivity to palbociclib.** (a) The qRT-PCR was performed to detect miR-29b-3p expression in MDA-MB-231, Hs578t, SK-BR-3 and MCF-7 cells. (b)The qRT-PCR was performed to detect miR-29b-3p expression after miR-29b-3p mimics transfected in MDA-MB-231 and Hs578t cells. (c) The qRT-PCR was performed to detect miR-29b-3p expression after miR-29b-3p inhibitor transfected in SK-BR-3 and MCF-7 cells. (d, e) Migration assays were performed in MDA-MB-231 and Hs578t cells after transfected with miR-29b-3p mimics or miR-29b-3p NC, or SK-BR-3 and MCF7 cells transfected with miR-29b-3p inhibitor or miR-29b-3p.

**Supplementary figure 5 related to figure 5. miR-29b-3p negatively regulates CDK6 expression.** (a) The qRT-PCR and (b) western blot analysis were performed to detect CDK6 expression after sh-CDK6-mediated knockdown in SK-BR-3 and MCF-7 cells and plasmid-medicated overexpression in MDA-MB-231 and Hs578t cells. (c) Western blot analysis and (d) migration assays were performed to detect CDK6 expression after transfection or treatment as indicated in the four breast cancer cells.
