# Supplementary figures and images for: c-myc regulates the sensitivity of breast cancer cells to palbociclib via c-myc/miR-29b-3p/CDK6 axis
Source: Cell Death Dis. 2020 Sep 15;11(9):760. doi: 10.1038/s41419-020-02980-2 (PMC7493901; doi:10.1038/s41419-020-02980-2)

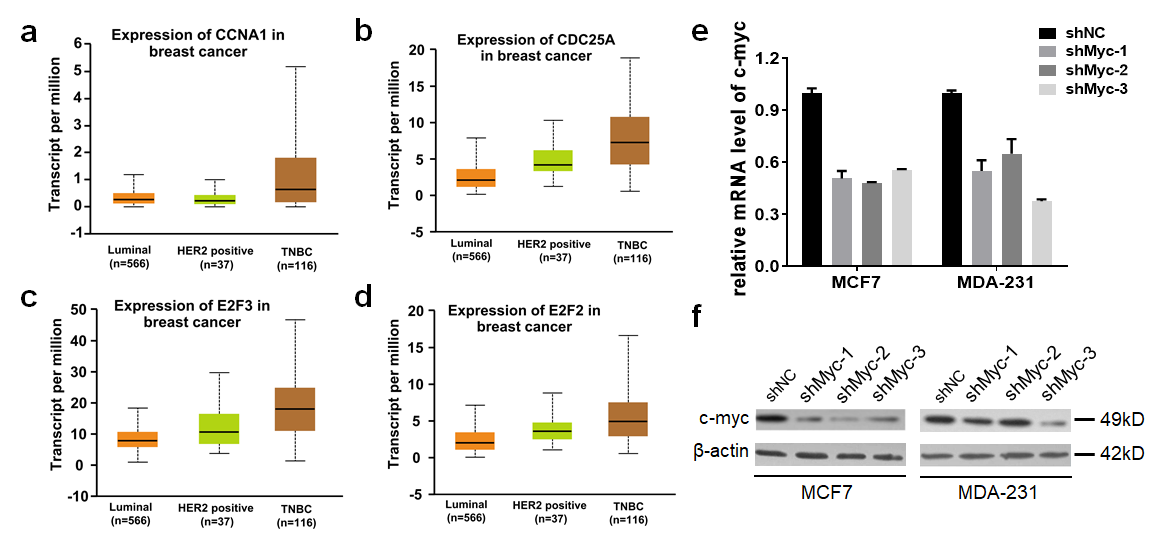

Supplement: Supplementary file 2 — Supplementary figure 1 [file 41419_2020_2980_MOESM2_ESM.tif]

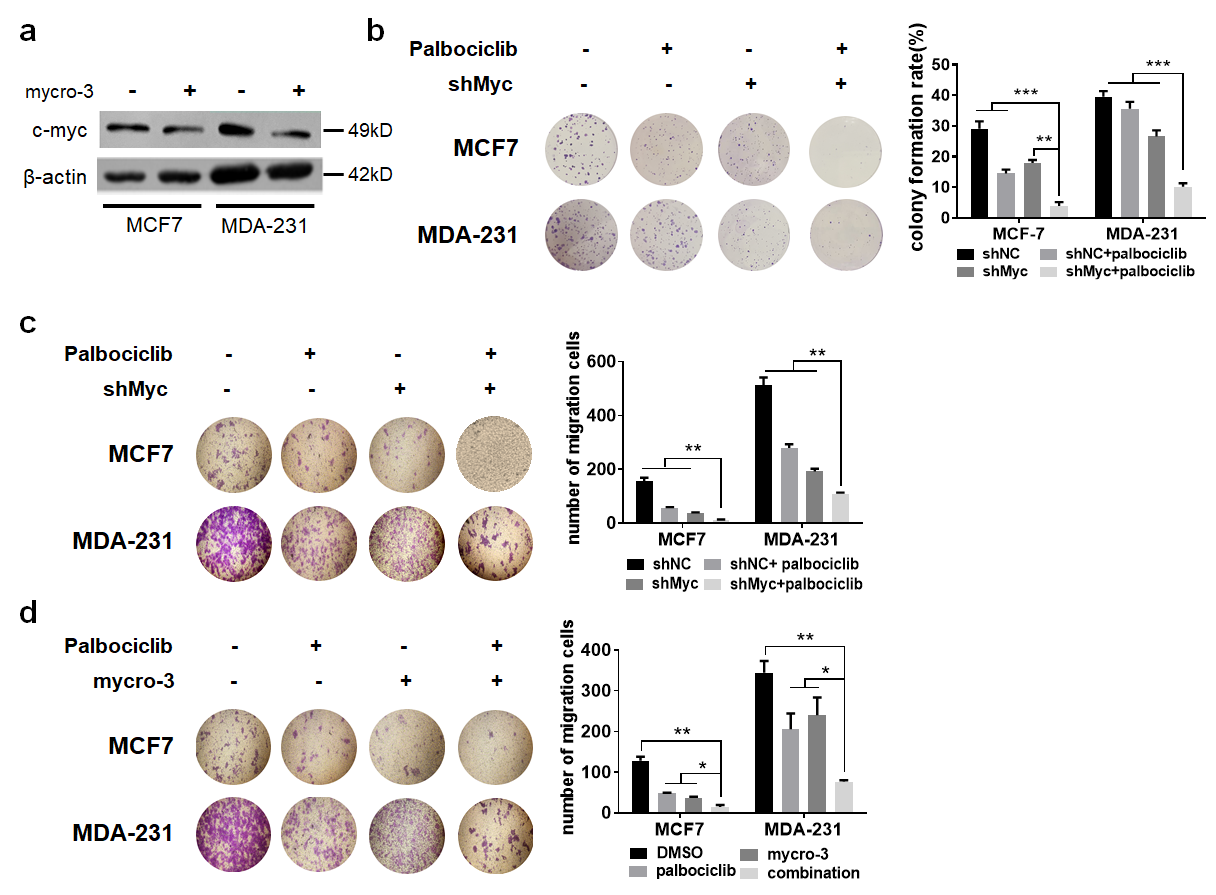

Supplement: Supplementary file 3 — Supplementary figure 2 [file 41419_2020_2980_MOESM3_ESM.tif]

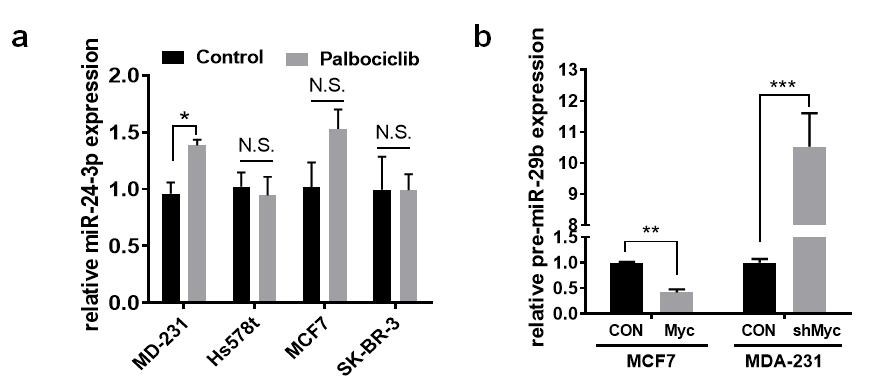

Supplement: Supplementary file 4 — Supplementary figure 3 [file 41419_2020_2980_MOESM4_ESM.tif]

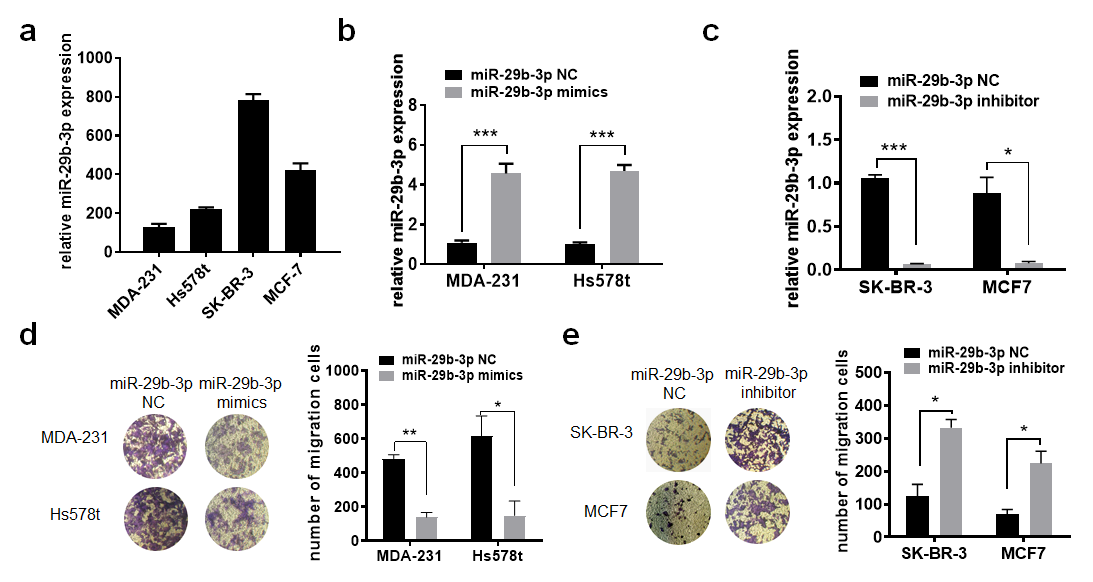

Supplement: Supplementary file 5 — Supplementary figure 4 [file 41419_2020_2980_MOESM5_ESM.tif]

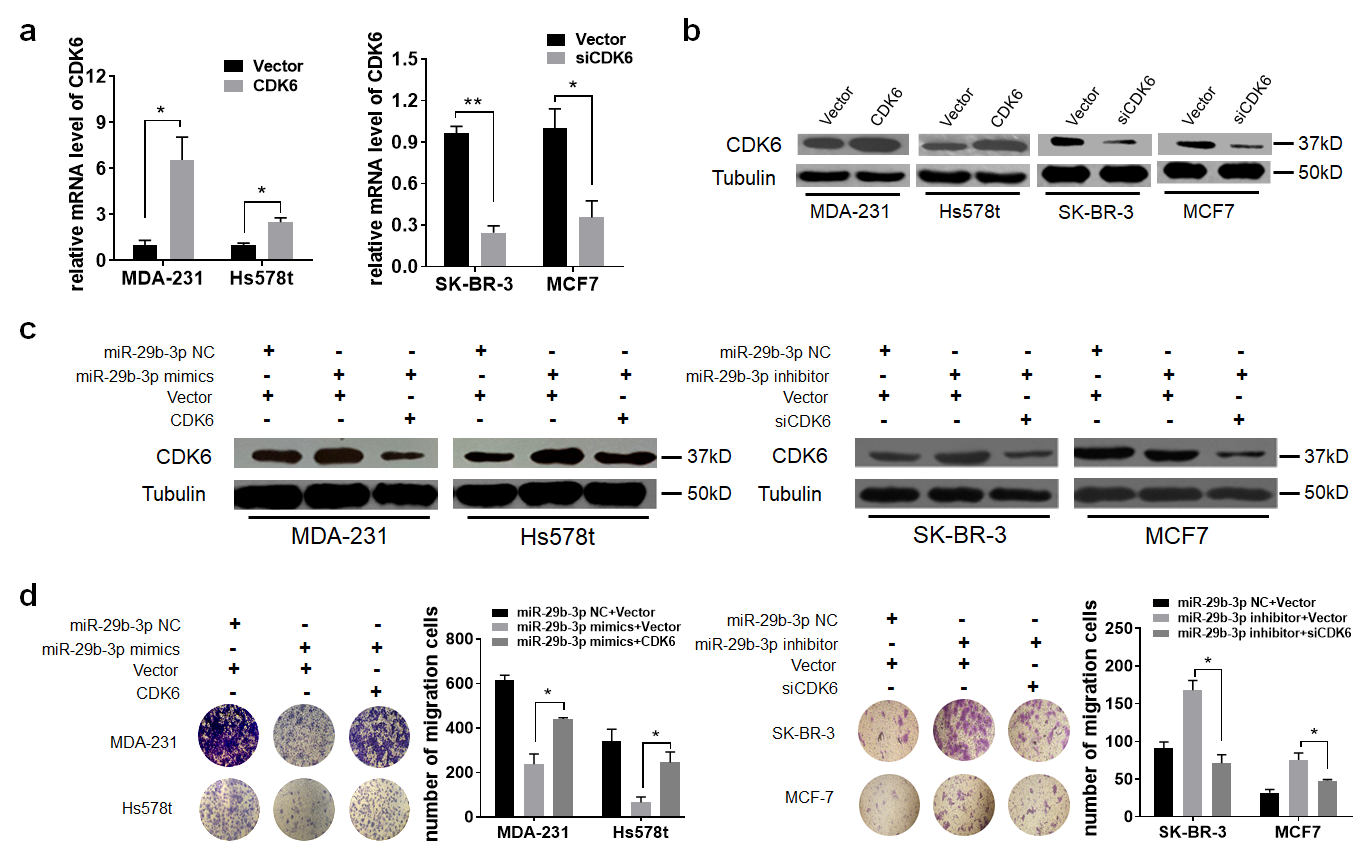

Supplement: Supplementary file 6 — Supplementary figure 5 [file 41419_2020_2980_MOESM6_ESM.tif]
